# The effectiveness of workplace dietary interventions: protocol for a systematic review and meta-analysis

**DOI:** 10.1186/s13643-016-0200-1

**Published:** 2016-02-03

**Authors:** Sarah A. Smith, Amelia A. Lake, Carolyn Summerbell, Vera Araujo-Soares, Frances Hillier-Brown

**Affiliations:** Fuse, UKCRC Centre for Translational Research in Public Health, Newcastle, UK; School of Medicine, Pharmacy and Health, Durham University, Queen’s Campus, Stockton-On-Tees, UK; Institute of Health and Society, Newcastle University, Newcastle-upon-Tyne, UK

**Keywords:** Workplace, Diet, Nutrition, Intervention, Health promotion, Systematic Review, Meta-analysis

## Abstract

**Background:**

The lack of evidence of the role of workplaces as settings for behaviour change delivery and the failure to recognise and address the complexity of the work environment has been acknowledged. This systematic review and meta-analysis will identify the effectiveness of dietary interventions in the workplace facilitating an understanding of what works, why and how by identifying key components of and examining the theoretical models of behaviour change underpinning successful dietary interventions in the workplace.

**Methods/design:**

We will conduct searches in MEDLINE, EMBASE, CINAHL, PsycINFO, CENTRAL and PubMed for studies that assess dietary interventions based within workplace settings in any country, of any length of time or duration of follow-up. We will include all randomised controlled trials (RCTs), non-randomised controlled trials (NRCTs), controlled before-after studies (CBAs) and interrupted time series (ITS) studies with a control group. Risk of bias of included studies will be assessed using a tool adapted from the Cochrane Public Health Review Group’s recommended Effective Public Health Practice Project Quality Assessment Tool for Quantitative Studies. Meta-analysis will be conducted if appropriate, or a narrative synthesis will be conducted following the ESRC Narrative Synthesis Guidance.

**Discussion:**

This paper outlines the study protocol for a systematic review and meta-analysis that will identify, critically appraise, and summarise the relevant evidence on the effectiveness and implications of interventions to promote healthier dietary behaviours in the workplace. This review will give an overview of the evidence and provide a guide for development of interventions promoting dietary behaviour change in workplaces.

**Systematic review registration:**

PROSPERO CRD42015015175

**Electronic supplementary material:**

The online version of this article (doi:10.1186/s13643-016-0200-1) contains supplementary material, which is available to authorized users.

## Background

The increasing prevalence of adults who are overweight and obese is continuing to pose a major global public health problem. Recent WHO global estimates show that overall, about 13 % of the world’s adult population aged 18 years and over (11 % of men and 15 % of women) were obese in 2014, and 39 % of adults (38 % of men and 40 % of women) were overweight [1]. In the UK, the proportion of obese adults has increased to 26 % in men and 23.8 % for women between 1993 and 2013 [2]. In the UK in 2010, on average, obese people took four extra sick days per year [3] which for the average company equates to more than £126,000 a year in lost productivity [4]. Estimates of the indirect costs of obesity such as loss of productivity in 2001 were £15.8 billion [5]. Coupled with the rise in obesity-associated comorbidities, the financial cost of obesity is continuing to rise.

In response to the rising global trend in obesity and overweight, WHO has developed the “Global Action Plan for the prevention and control of non-communicable diseases 2013–2020” [1] which aims to build on the WHO Framework Convention on Tobacco Control and the WHO Global Strategy on Diet, Physical Activity and Health. The plan will contribute to nine global targets to be attained in 2025, including a halting of the global obesity rates to those of 2010. There is a global need to develop and evaluate dietary interventions conducted in various settings to address the ‘globesity’ problem [6, 7]. Amongst others, the workplace environment has been identified as an ideal setting for health interventions [8] in which to tackle diet and lifestyle behaviours [9]. Workplace interventions have the potential to target a large proportion of the adult population particularly as those in employment can spend up to two thirds of their day at work [10–14]. Furthermore, prevalence of overweight and obesity varies by age, with higher prevalence in older age groups amongst both men and women [1]. With an aging population and a greater proportion of people working past retirement age, the positive impact of workplace interventions could be seen across the working lifespan. The scope of workplace interventions to address overweight and obesity is great, with the potential to impact on individuals across society; however, the greatest benefit may be for those in full-time employment who can access onsite catering and interventions which are typically delivered during daytime working hours. Those who work part-time, particularly women, or during evenings and during the night may not have the same access as full-time workers; therefore, there is the risk of creating health inequalities across organisations. There is a need for more studies of the effectiveness of interventions in reducing inequalities in obesity for both men and women, with an emphasis on macro- or organisational-level interventions, such as workplaces, that have the potential to address the entire gradient [15].

Despite the obvious potential of workplace interventions targeting health behaviours, few UK-based workplace intervention studies have been published. Fewer still focus on the practicalities and implications when running an intervention within the workplace setting [16], and there is still uncertainty about the effectiveness of dietary interventions in the workplace. A number of workplace-based interventions have attempted to change dietary behaviour [17–21]. Techniques such as education, counselling and alterations to the physical environment of the workplace have all been used in an attempt to modify dietary intake [22, 23]. A number of systematic reviews into workplace interventions have shown that environmental modifications and education in relation to diet, physical activity, and lifestyle factors have, in general, lead to moderate improvement in dietary intake [24–26]. However, the lack of evidence regarding the role of worksites and in particular the failure of many interventions to recognise and address the complexity of the work environment has been acknowledged.

This systematic review of published controlled trials will identify, critically appraise and summarise the relevant evidence on the effectiveness and implications of interventions to promote healthier dietary behaviours in the workplace. Furthermore, this review will facilitate an understanding of what works, why and how by identifying key components of and examining the theoretical models of behaviour change underpinning successful dietary interventions in the workplace. The findings will provide a guide for the development of future interventions and research into tackling workplace obesogenic environments and promoting positive dietary behaviour change in workplaces.

## Objectives

The objectives are as follows:

1. To identify what workplace-based interventions are effective at reducing energy intake, reducing fat intake, reducing salt intake and reducing consumption of sugar sweetened beverages and/or sweets.
2. To identify what workplace interventions are effective at increasing fruit and/or vegetable consumption and/or increasing fibre intake.
3. To identify what workplace interventions are effective at reducing and/or controlling food portion size.
4. To explore if some subgroups of the population are more responsive to such interventions (for example, older versus younger employees, men versus women, shift workers versus non-shift workers, night shift workers, manual versus professional, socioeconomic status, ethnicity).
5. To explore if changes in weight, body mass index, body composition and/or waist circumference are observed in response to dietary interventions in the workplace.
6. To explore if changes in employee wellbeing, productivity and absenteeism are observed in response to dietary interventions in the workplace.
7. The interventions included in this systematic review will be analysed, as much as possible, on the basis of the behaviour change techniques used.

## Methods/design

Prior to conducting the review, we carried out a scoping search to ensure adequate sensitivity of the search strategy. The search was piloted in MEDLINE (searched 27 April 2015) and resulted in 2069 hits. Furthermore, five indicator papers identified prior to running the search were included in the results [27–31] suggesting we would find sufficient evidence from controlled studies to meet our objectives. Therefore, we will only include evidence from randomised controlled trials (RCTs), non-randomised controlled trials (NRCTs), controlled before-after studies (CBAs) and interrupted time series (ITS) studies with a control group. Cochrane Effective Practice and Organisation of Care (EPOC) [32] study design criteria will used to identify studies. The review is registered with PROSPERO (International prospective register of systematic reviews) at the National Institute for Health Research and Centre for Reviews and Dissemination (CRD) at the University of York (registration number CRD42015015175) and will be reported in accordance with the PRISMA [33, 34] statement and MECIR standards [35]. Additional files show the PRISMA statement and checklist in more detail [see Additional file 1 and Additional file 2].

## Population

The studied population will be adults of all gender, socioeconomic status and nationality, with a mean age of 16 or older who are employed at the worksite.

## Interventions

The review will examine interventions that will target dietary behaviours that are based in any workplace in any country. The types of interventions expected to be found include, but are not restricted to, educational, environmental changes, counselling and food provision. There will be no restriction on the length of the intervention, but only studies with duration (intervention plus follow-up) of 4 weeks or over will be included. This criterion is set so as to be as inclusive as possible of studies, and to study the short-term as well as long-term effects of interventions. Workplace dietary interventions of 4 weeks have been shown to be effective at changing behaviour and sometimes resulting in weight change [36–39].

## Comparator

Studies with a comparator will be included in the review. There will be no restrictions on the type of comparator used in the study.

## Outcomes

Studies will be included if they have at least one primary outcome of interest. This includes a dietary intake outcome (change in vegetable consumption (g or SV). change in fruit consumption (g or SV), change in fruit and vegetable consumption (g or SV), change in consumption of sugar sweetened beverages (g), change in consumption of ‘other foods’, catering/food sales data) and a nutritional intake outcome (change in energy intake (kcal), change in fat intake (g), change in salt intake (g), change in fibre intake (mmHg), change in portion size (g or SV), change in food environment). The review will include various methods of outcome measurement, for example, but not restricted to, self report, researcher observations, photographs of food portions and weighed intake.

Data on related secondary outcomes (such as change in weight (kg), change in body mass index (kg/m^2^), change in body composition, change in waist circumference (cm) and improvement in productivity and reduction in absenteeism) will also be extracted from those studies which have a primary outcome. Where reported, we will include data on differential effects between specific populations (for example, older versus younger employees, men versus women, shift workers versus non-shift workers, night shift workers, manual versus professional, socioeconomic status, ethnicity).

## Literature searching

We will run one overarching search (amended to suit syntax requirements) to identify studies of relevance, and the electronic databases to be searched include MEDLINE (Ovid), EMBASE (Ovid), CINAHL (Ebscohost), PsycINFO (Ebscohost), CENTRAL (Cochrane Central Register of Controlled Trials) and PubMed. An additional file shows the search strategy in more detail [see Additional file 3]. All databases will be searched from inception to present day as of 11 May 2015.

## Searching other sources

Reference lists of included studies and relevant systematic reviews will be searched for any additional papers not picked up by our database searches. Known topic experts in a variety of countries will be contacted via email to enquire of any additional interventions they are aware of that may be of relevance to this review.

## Study selection and screening

A reviewer (SS) will independently screen the titles and abstracts to identify those that are relevant and meet the inclusion criteria. A second reviewer (FH, AL) will screen a random 10 % sample of titles and abstracts. Full texts of each included paper will be obtained and reviewed by one reviewer (SS) to determine which papers to include in the review, based on the inclusion and exclusion criteria. A second reviewer (FH, AL) will screen a random 10 % sample of the full texts. In the situation where first and second reviewers cannot decide on inclusion or exclusion of a title/abstract/paper, a third reviewer (CS, VA) will be consulted to achieve consensus.

## Data extraction

Data extraction will be conducted independently for each study by two reviewers (SS, FH, AL, VA, CS). Electronic data extraction forms (pre-established to ensure consistency and accuracy between reviewers) will be manually completed and will include details on study design, participant characteristics, intervention design, intervention duration, study methods, study outcomes, theory underpinning intervention design [40] (utilising the Behaviour Change Wheel [41] and the Nuffield Intervention Ladder [42]), economic cost (purchasing patterns, productivity, absenteeism), funding source and quality assessment. To improve the usefulness of the review findings for policy decision making, every attempt will be made to report cost and cost-effectiveness, study characteristics, and generalisability of the findings [43]. Any discrepancies will be resolved by discussion between the two reviewers, and if a decision is not met, a third reviewer will be consulted to reach consensus. The data extraction form will be piloted using a sample of studies and amendments made if required, then a second phase of pilot testing conducted.

## Assessment of risk of bias (quality) in included studies

To assess the quality of each study, a tool adapted from the Cochrane Public Health Review Group’s recommended Effective Public Health Practice Project Quality Assessment Tool for Quantitative Studies [44] will be used. The tool includes representativeness of study samples, randomisation, comparability of baseline groups, credibility of data collection tools, attrition rate and attributability to the intervention. As before, any discrepancies will be resolved by discussion between the two reviewers, and if a decision is not met, a third reviewer will be consulted to reach consensus.

## Data analysis and synthesis

We will report our findings in accordance with PRISMA guidelines [33, 34]. A narrative synthesis is planned and will be conducted following the ESRC Narrative Synthesis Guidance [45], but if more than one study is included and the studies are adequately similar in terms of study populations, interventions comparators and outcome investigated, it is intended that a meta-analysis will be preformed. Data synthesis will be carried out in Review Manager (Cochrane Collaboration Software), and both continuous and dichotomous data will be analysed. If high heterogeneity does not exist, we will carry out fixed-effect analysis. If important heterogeneity exists, we will carry out random-effect analysis.

## Analysis of subgroups or subsets

If there are sufficient data, a sub-group analysis will be conducted. Subgroups proposed are interventions conducted in different workplaces, different occupations (manual, professional), various types of dietary interventions (including but not limited to increased fruit and/or vegetable consumption, portion size control, environmental changes), age (less than 30 years and more than 30 years), gender (men versus women), shift workers versus non-shift workers, professional versus manual workers, socioeconomic status and ethnicity.

## Discussion

This review aims to summarise the evidence on the effectiveness of interventions targeting dietary intake in workplaces. It will explore if, and how, factors such as age, gender, socioeconomic status, ethnicity, working patterns and job hierarchy moderate the effects of the interventions. It will also explore if, and how, dietary workplace interventions impact health in terms of changes in body weight and/or body composition and impact worker productivity and absenteeism rates. The review will describe the included interventions in terms of how they are designed, implemented and delivered as well as attempt to discuss the policy and practice implications of the findings, which will be useful in guiding future development of interventions and research into tackling workplace obesogenic environments and promoting positive dietary behaviour change in workplaces.

## Additional files

Additional file 1:
**PRISMA checklist.** The full PRISMA statement checklist for systematic reviews. (PDF 562 kb) Additional file 2:
**PRISMA flow diagram.** The full PRISMA statement flow diagram for systematic reviews. (PDF 332 kb) Additional file 3:
**Search strategy.** The complete MEDLINE version of the search strategy. (PDF 281 kb)

## Abbreviations

CENTRAL: Cochrane Central Register of Controlled Trials
CINAHL: Cumulative Index to Nursing and Allied Health Literature
CRD: National Institute for Health Research and Centre for Reviews and Dissemination, York
Ebscohost: provides access to bibliographic databases designed for research
EMBASE: Excerpta Medica database
EPOC: Cochrane Effective Practice and Organisation of Care
ESRC: The Economic and Social Research Council
Fuse: the Centre for Translational Research in Public Health
MECIR: Methodological Expectations of Cochrane Intervention Reviews
MEDLINE: Medical Literature Analysis and Retrieval System Online
Ovid: provides access to online bibliographic databases and academic journals
PRISMA: Preferred Reporting Items for Systematic Reviews and Meta-Analyses
PROSPERO: International prospective register of systematic reviews
PsycINFO: a database of abstracts of literature in the field of psychology
PubMed: a free search engine for literature
UKCRC: UK Clinical Research Collaboration
WHO: World Health Organisation
