# Supplementary material for: The effectiveness of workplace dietary interventions: protocol for a systematic review and meta-analysis
Source: Syst Rev. 2016 Feb 3;5:20. doi: 10.1186/s13643-016-0200-1 (PMC4740991; doi:10.1186/s13643-016-0200-1)
Supplement: Additional file 2: — PRISMA flow diagram. The full PRISMA statement flow diagram for systematic reviews. (PDF 332 kb) [file 13643_2016_200_MOESM2_ESM.pdf]

PRISMA 2009 Flow Diagram

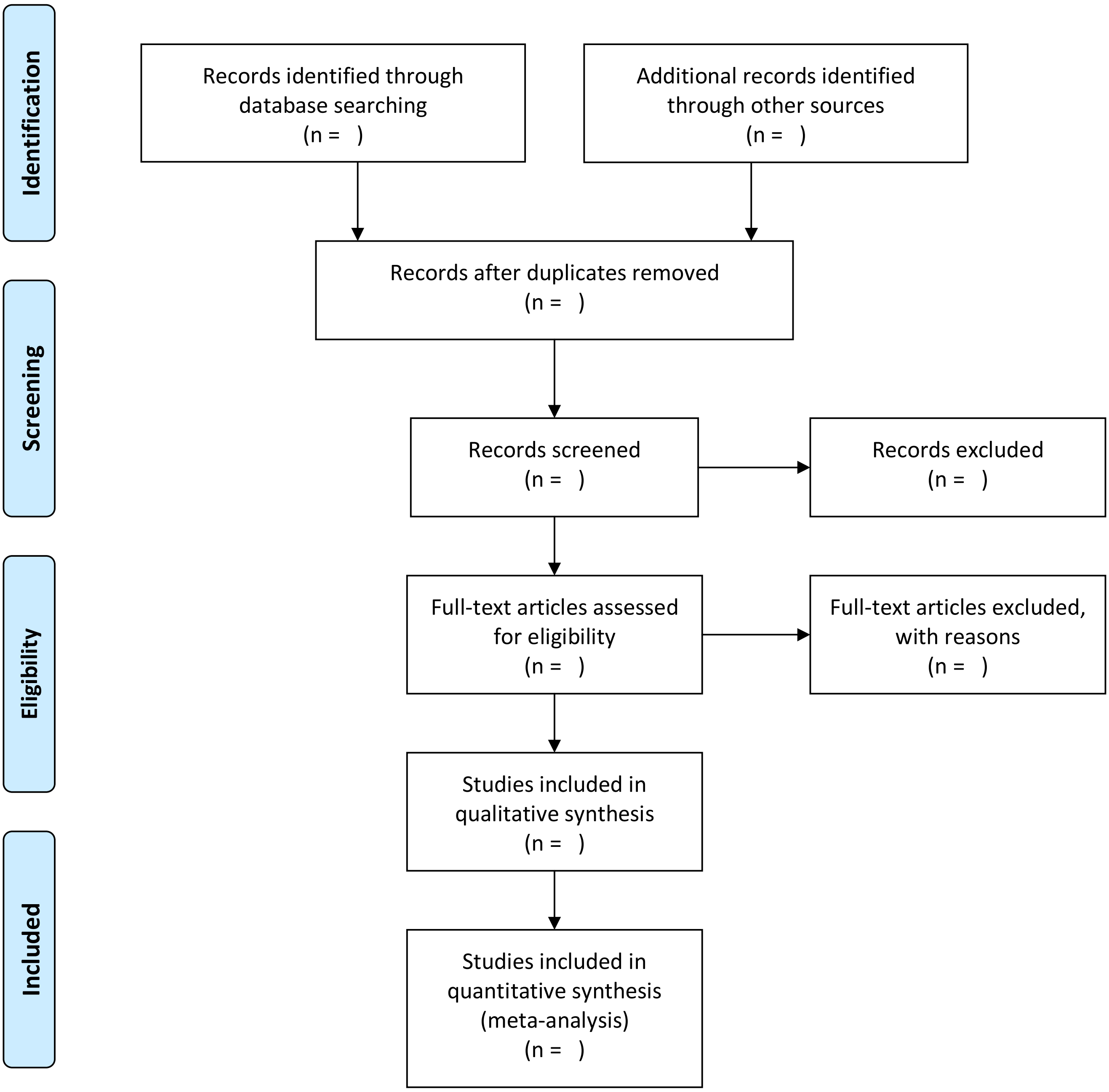

From: Moher D, Liberati A, Tetzlaff J, Altman DG, The PRISMA Group (2009). *P*referred *R*eporting *I*tems for *S*ystematic Reviews and *M*eta-*A*nalyses: The PRISMA Statement. PLoS Med 6(6): e1000097. doi:10.1371/journal.pmed1000097

For more information, visit www.prisma-statement.org.
