# Supplementary material for: The effectiveness of workplace dietary interventions: protocol for a systematic review and meta-analysis
Source: Syst Rev. 2016 Feb 3;5:20. doi: 10.1186/s13643-016-0200-1 (PMC4740991; doi:10.1186/s13643-016-0200-1)
Supplement: Additional file 3: — Search strategy. The complete MEDLINE version of the search strategy. (PDF 281 kb) [file 13643_2016_200_MOESM3_ESM.pdf]

Additional File 3: Search strategy for MEDLINE

1. employee$.ti,ab.

2. worker$.ti,ab.

3. (workforce$ or work force$).ti,ab.

4. *work/

5. *workplace/

6. (workplace$ or work place$).ti,ab.

7. (worksite$ or work site$).ti,ab.

8. (workfloor$ or work floor$).ti,ab.

9. (work adj2 environment).ti,ab.

10. *employment/

11. 1 or 2 or 3 or 4 or 5 or 6 or 7 or 8 or 9 or 10

12. *food/

13. food$.ti,ab.

14. exp diet/

15. diet$.ti,ab.

16. nutri$.ti,ab.

17. fruit/

18. fruit$.ti,ab.

19. vegetables/

20. vegetable$.ti,ab.

21. energy.ti,ab.

22. fat$.ti,ab.

23. *dietary fats/

24. salt$.ti,ab.

25. fibre$.ti,ab.

26. (sugar$ adj2 sweetened$ adj2 beverages$).ti,ab.

27. (portion$ adj2 size$).ti,ab.

28. (serving$ adj2 size$).ti,ab.

29. cafeteria$.ti,ab.

30. canteen$.ti,ab.

31. (healthy adj2 eating).ti,ab.

32. (catering$ adj2 service$).ti,ab.

33. (catering$ adj2 establishment).ti,ab.

34. (food$ adj2 service$).ti,ab.

35. exp food services/

36. (food$ adj2 environment$).ti,ab.

37. 12 or 13 or 14 or 15 or 16 or 17 or 18 or 19 or 20 or 21 or 22 or 23 or 24 or 25 or 26 or 27 or 28 or 29 or 30 or 32 or 32 or 33 or 34 or 35 or 36

38. 11 and 37

39. intervention$.ti,ab.

40. *intervention studies/

41. campaign$.ti,ab.

42. program$.ti,ab.

43. (strategy$ or strategies$).ti,ab.

44. award$.ti,ab.

45. scheme$.ti,ab.

46. promotion$.ti,ab.

47. exp health promotion/

48. 39 or 40 or 41 or 42 or 43 or 44 or 45 or 46 or 47

49. 38 and 48

50. (randomized controlled trial OR controlled clinical trial).pt.

51. randomized controlled trials/

52. random allocation/

53. double-blind method/

54. single-blind method/

55. 50 or 51 or 52 or 53 or 54

56. animal/ not human/

57. 55 not 56

58. clinical trial.pt.

59. clinical trials/

60. (clinic$ adj25 trial$).tw.

61. ((singl$ or doubl$ or trebl$ or tripl$) adj (mask$ or blind$)).tw.

62. Random$.tw.

63. Research Design/

64. (latin adj square).tw.

65. 58 or 59 or 60 or 61 or 62 or 63 or 64

66. 65 not 56

67. 66 not 57

68. Comparative study/

69. Evaluation studies/

70. Follow-up studies/

71. Prospective studies/

72. (control$ or prospective$ or volunteer$).tw.

73. Cross-over studies/

74. 68 or 69 or 70 or 71 or 72 or 73

75. 74 not 56

76. 75 not (57 and 67)

77. 57 or 67 or 76

78. Controlled study/

79. (Controlled Trial or non-randomised controlled trial).ti,ab.

80. Controlled adj (before and after study).ti,ab.

81. ((before and after study) adj10 control).ti,ab.

82. (Interrupted Time Series adj10 control).ti,ab.

83. 78 or 79 or 80 or 81 or 82

84. 83 or 77

85. 49 and 84

86. limit 85 to (humans)
